# Clinical value of ^18^F-FDG PET/CT in the management of HIV-associated lymphoma

**DOI:** 10.3389/fonc.2023.1117064

**Published:** 2023-01-26

**Authors:** Qi Liu, Tao Yang, Xiaoliang Chen, Yao Liu

**Affiliations:** ^1^ Department of Nuclear Medicine, Chongqing University Cancer Hospital, Chongqing, China; ^2^ Department of Hematology-Oncology, Chongqing University Cancer Hospital, Chongqing, China

**Keywords:** HIV, diffuse large B-cell lymphoma, Burkitt’s lymphoma, Hodgkin’s lymphoma, ^18^F-FDG PET

## Abstract

HIV is still a major public health problem. At present, HIV-associated lymphoma remains the leading cause of deaths among people living with HIV, which should be paid more attention to. ^18^F-fluorodeoxglucose (FDG) PET/CT has been recommended in the initial staging, restaging, response assessment and prognostic prediction of lymphomas in general population. HIV-associated lymphoma is, however, a different entity from lymphoma in HIV-negative with a poorer prognosis. The ability to accurately risk-stratify HIV-infected patients with lymphoma will help guide treatment strategy and improve the prognosis. In the review, the current clinical applications of ^18^F-FDG PET/CT in HIV-associated lymphoma will be discussed, such as diagnosis, initial staging, response evaluation, prognostic prediction, PET-guided radiotherapy decision, and surveillance for recurrence. Moreover, future perspectives will also be presented.

## 1 Introduction

Acquired immunodeficiency syndrome (AIDS) is a major disease that greatly endangers human health across the world. AIDS is caused by human immunodeficiency virus (HIV), which progressively destroys CD4 + T cells and compromises the function of the immune system leading to severe opportunistic infection or cancer (1). HIV infection is associated with an increased risk of a range of cancers, which are classified into two groups: AIDS-defining cancers(ADCs) and non-AIDS-defining cancers (NADCs) (2). There are three ADCs: Kaposi sarcoma (KS), non-Hodgkin lymphoma (NHL), and cervical cancer. The incidence of NHL exceeded KS in 2018 and became the highest ADC according to the statistics of American National Cancer Institute, which was 1194 and 765 cases/year, respectively (2). With the exception of the three ADCs, all cancers in HIV-infected population are considered as NADCs, including HIV-associated tumors and incidental cancers. The most common types of NADCs found in persons living with HIV include those with a higher rate among HIV-infected population (such as anal cancer, liver cancer, oropharyngeal cancer, Hodgkin lymphoma and lung cancer, which compose about half of all cancers among HIV-infected persons in the United States) and those common in the general population (such as prostate, breast, and colon cancer) (2, 3). In a meta-analysis of 4,797 NADC cases, the most common NADC type was lung cancer (17.6%), followed by Hodgkin lymphoma (13.4%), anal cancer (5.3%) and liver cancer (3.5%) (4). With the advent of the highly active antiretroviral therapy (HAART) era, the standard incidence rate of NHL has dropped dramatically, whereas the standard incidence rate of HL increased (5, 6). At present, HIV-associated lymphoma remains the leading cause of deaths among people living with HIV, which should be paid more attention to (7, 8).

Diffuse large B-cell lymphoma (DLBCL) and Burkitt’s lymphoma (BL) are among the most common subtypes of NHL in ADC in the HAART era (2, 9), with an incidence of 1.20 and 0.32 per 1000 person-years, respectively (9, 10). Other rare forms of NHL, including plasmablastic lymphoma (PBL), primary effusion lymphoma (PEL), and primary central nervous system lymphoma (PCNSL), are also associated with HIV infection (11–13). Furthermore, compared to HIV-negative persons, the risk of HL which is not AIDS-defining is higher among HIV-infected persons (3).

Previous studies have shown that ^18^F-fluorodeoxglucose (FDG)-positron emission tomography (PET)/computed tomography (CT) plays an important role in management of HIV-negative patients with lymphoma (14, 15). This noninvasive technology provides several quantitative metabolic parameters such as the maximum of standard uptake value (SUVmax), SUV at lean body mass (SUL), metabolic tumor volume (MTV), and total lesion glycolysis (TLG) for the management of lymphoma. Due to high sensitivity to whole-body involved lymph nodes and extranodal lymphoma, ^18^F-FDG PET/CT has been incorporated into standard assessment for all FDG-avid lymphomas, and is recommended by the 2014 Lugano criteria (16). Meanwhile, several large randomized clinical trials demonstrated that, decisions to reduce or increase the intensity of treatment can be based on the changes in tumor metabolism during lymphoma treatment (17–19).

However, HIV-associated lymphoma is a different entity from lymphoma in the general population. Compared with lymphoma in HIV-negative persons, HIV-associated lymphoma has a worse prognosis, more frequent occurrence of B symptoms (i.e., weight loss, night sweats, and fever) and more advanced Ann Arbor stages, even in the HAART era (20, 21). HIV-related benign lymphadenopathy is a main reason for a false positive of HIV-associated lymphoma on FDG PET. Whether HIV-infected status will increase tumor burden and reduce overall survival is still under discussion. To date, there are relatively few studies on the clinical value of FDG PET in HIV-associated lymphoma.

This article presents an overview of current ^18^F-FDG PET/CT clinical applications in the management of patients with HIV-associated lymphoma, including initial staging, response evaluation, prognostic prediction, PET-guided radiotherapy decision and differentiated diagnosis. Furthermore, future perspectives will also be discussed.

## 2 PET to distinguish lymphoma from benign lesions

### 2.1 HIV-associated lymphoma vs. benign lymphadenopathy

Lymphadenopathy in HIV-infected patients is a diagnostic challenge for nuclear medicine physicians and clinicians. HIV has a strong tropism for lymphoid tissues, and it leads to a progressive alteration in lymph nodes architecture that clinically manifests as persistent lymphadenopathy in three histologic phases: stage I, explosive follicular hyperplasia with large germinal centers; stage II, germinal center involution and lymphocytes depletion; stage III, proliferation of blood vessels inside the lymph nodes (22). In addition to the HIV infection itself, the other main causes of lymphadenopathy include HIV-associated lymphoma, opportunistic infections, and inflammatory conditions (23–25). Immune reconstitution inflammatory syndrome (IRIS) should also be considered as a possible cause of lymphadenopathy in patients who start HAART. The incidence of lymphadenopathy is negatively correlated with CD4 counts (26). Due to the different respective treatments and adverse effects of over treatment, it is vital to distinguish HIV-associated lymphoma from benign lymphadenopathy.

A qualitative analysis assessed the role of PET/CT in differentiating NHL from benign lymphadenopathy in patients with HIV-1. In 16 PET/CT scans, PET/CT accurately depicted the context of lymphoma. 12 patients with concordant PET/CT (+/+) findings had well-suppressed viral loads and low CD4 levels, whereas the 4 PET/CT (+/−) scans had the highest values of both laboratory parameters. In the case of inconsistent PET and CT findings, increased viral loads and CD4 count may imply benign lymphadenopathy (27). Since the advent of HAART, PET/CT (+/−) findings concurrent with a rapid increase of CD4 count may imply benign lymphadenopathy, perhaps due to IRIS (28, 29). In acute phase of HIV, FDG-avid lymph nodes are typically observed in the head and neck. In late phase, involvement of abdominal lymph nodes is more common (30). In addition, Liu et al. showed that concurrent nasopharyngeal lesions and lymphadenopathy on PET/CT had a greater possibility of malignant lymphoma instead of benign lymphoproliferative disease or nasopharyngeal carcinoma (31).

In quantitative analysis of ^18^F-FDG PET/CT, Chen et al. identified that the SUVmax of only lymph nodes (SUV_LN_), and the most FDG-avid lesion to liver SUVmax ratio (SURmax) provided a new basis for distinguishing malignant lymphoma from inflammatory lymphadenopathy in patients with HIV. This study retrospectively assessed 59 HIV-infected patients, of which 37 had HIV-associated lymphoma, and 22 had HIV-associated inflammatory lymphadenopathy. Malignant lymphoma invaded more commonly to extra-lymphatic lesions, compared to inflammatory lymphadenopathy (83.8% vs. 54.5%, p=0.000). Especially, the involved lesions of digestive tract and Waldeyer’s ring obviously differed between the two groups (p=0.004 and 0.033, respectively). Furthermore, the SURmax, SUV_LN_, SUV_Marrow_ and SUV_Liver_ in malignant lymphoma were significantly higher than those in inflammatory lymphadenopathy (p=0.000, 0.000, 0.002 and 0.017, respectively). The cut-off point of 3.1 for SURmax showed the best equilibrium between sensitivity (68.2%) and specificity (91.9%), and the cut-off point of 8.0 for the SUV_LN_ had also high specificity (89.2%) and relatively reasonable sensitivity (63.6%). The cut-off point of 5 for numbers of involved areas and 3.6 centimeter for maximum diameters of lymph node had relatively low sensitivity (62.2%, 64.9%, respectively) and specificity (72.7%, 86.4%, respectively) (32).

Another retrospective study assessed the diagnostic accuracy of the quantitative PET indices for distinguishing HIV-associated lymphoma from reactive lymphadenopathy, including MTV, TLG, maximum and peak of SUL (SULmax and SULpeak). All of the quantitative PET metrics were significantly higher in patients with lymphoma than in those with reactive lymphadenopathy (all p values, <0.001). The cut-off points of 173 for TLG and 53.8 for MTV showed high sensitivity (89%, 84%, respectively) and specificity (100%, 100%, respectively) for differentiating lymphoma from reactive lymphadenopathy. A summed SULpeak cut-off point of 23.8 showed a sensitivity and specificity of 84% and 95% respectively, whereas a summed SULmax cut-off point of 28.4 yielded a sensitivity and specificity of 84% and 82% respectively. In the reactive lymphadenopathy group, PET metrics were positively correlated with viral load. However, in the HIV-associated lymphoma group, none of the PET parameters showed a significant correlation with viral load. In the qualitative MIP symmetry score analysis of the pattern of lymph node involvement, asymmetrical FDG uptake had an accuracy of 90.4% for differentiating lymphoma from reactive adenopathy in HIV-infected patients (33). The diagnostic efficacies of FDG PET/CT PET/CT in differentiating HIV-associated lymphoma from benign lymphadenopathy are summarized in **Table 1**.

**Table 1:** The diagnostic efficacies of FDG PET/CT in differentiating HIV-associated lymphoma from benign lymphadenopathy.

References	Number of patients	Subtypes of lymphoma	Parameters	Cut-off point	AUC	P value	Specificity (%)	Sensitivity (%)	PPV (%)	NPV (%)
32	59 (37 HAL)	35 B-cell lymphoma, 1 HL, 1 T-cell lymphoma	SURmax	3.1	0.888	**0.000**	91.9	68.2	NA	NA
			SUV_LN_	8.0	0.815	**0.000**	89.2	63.6	NA	NA
			SUV_Marrow_	NA	0.611	0.156	NA	NA	NA	NA
			SUV_Liver_	NA	0.567	0.393	NA	NA	NA	NA
			Number of lymph node involved areas	5	0.692	**0.014**	72.7	62.2	NA	NA
			Maximum diameter of lymph nodes	3.6	0.768	**0.001**	86.4	64.9	NA	NA
33	41 (19 HAL)	16 DLBCL, 3 HL	Single SULmax	7.8	0.971	NA	100	89	100	92
			TLG	173.0	0.964	NA	100	89	100	92
			Single SULpeak	6.6	0.964	NA	100	84	100	88
			MTV	53.8	0.957	NA	100	84	100	88
			Sum SULpeak	23.8	0.935	NA	95	84	94	88
			Sum SULmax	28.4	0.904	NA	82	84	80	86

Statistically significant values are highlighted in bold.

1. AUC, area under the curve; PPV, positive predictive value; NPV, negative predictive value; NA, not available; 2. DLBCL, diffuse large B-cell lymphoma; HL, Hodgkin’s lymphoma; 3. SUVmax, the maximum of standard uptake value; SURmax, liver SUVmax ratio; SUV_LN_, SUVmax of only lymph nodes; SUL, SUV at lean body mass; SULmax, the maximum of SUL; SULpeak, the peak of SUL; TLG, total lesion glycolysis; MTV, metabolic tumor volume.

### 2.2 PCNSL vs. cerebral opportunistic infection

PCNSL and cerebral toxoplasmosis (CTOX) are the two most common diagnoses of intracranial mass in HIV-infected patients. PCNSL is a rare extranodal NHL, with an incidence of 4-5/1000 among patients with HIV (34). The pooled prevalence of CTOX among HIV-infected patients is approximately 35.8% (35). It is still a challenge for conventional imaging to distinguish these two entities because of overlapping and non-specific imaging features (35, 36). Because of the poor prognosis and the fatal outcome of misdiagnosis or delayed treatment (37), accurate and prompt diagnosis is crucial.

In a prospective study of HIV-infected patients, Westwood et al. identified that foci of increased FDG uptake corresponding to the enhanced lesions on MRI was considered suggestive of PCNSL. Conversely, no clinically significant increase in FDG uptake was considered suggestive of CTOX. One patient had progressive multifocal leukoencephalopathy (PML) with equivocal metabolic activity, and the other one with hemorrhagic brain metastasis showed normal metabolic acitivity (38).

Hoffman et al. showed increased FDG uptake of PCNSL compared with benign lesions. For qualitative analysis, a visual scoring system was proposed. For semiquantitative assessment, a count ratio of lesion to contralateral homologous brain was calculated. Both qualitative visual scoring and count ratios (1.8,0.65, 1.3, and 0.7, respectively; p=0.006) suggested higher scores and count ratios in lymphoma than those in nonmalignant lesions (toxoplasmosis, PML, syphilis) (39). These results were supported by another semiquantitative study, in which the SUV ratio of the lesion to the contralateral brain in HIV-infected patients with PCNSL was higher than that in patients with cerebral infections including toxoplasmosis and tuberculoma (range, 1.7-3.1, 0.3-0.7, respectively; p<0.05) (40). A similar semiquantitative analysis suggested that PCNSL showed increased lesional uptake in comparison with the normal brain cortex (mean SUVmax, 18.8; range, 12.4-29.9), while CTOX had lesional uptake less than that of normal brain cortex (mean SUVmax, 3.5; range, 1.9-5.8) (41).

It should be noted that the challenge in applying semiquantitative analyses similar to the above methods arises when the corresponding contralateral brain exhibits physiologically increased FDG uptake. Nevertheless, delayed ^18^F-FDG PET/CT has been found to be effective in differentiating malignancy from inflammation or infection. Compared to benign lesions or normal tissues, malignant tumors show a progressive increase in metabolic activity, which have improved the background contrast with lesion-based sensitivity as high as 98% (42, 43).

## 3 PET for initial staging

As described above, HIV-positive patients are at higher risk of lymphoma, making the accurate appreciation of the extent of disease based on reliable initial staging imperative. The role of ^18^F-FDG PET/CT in initial staging of lymphoma is superior to contrast-enhanced CT, especially for the detection of lesions with no or minor anatomical abnormalities (including bone marrow, spleen and gastrointestinal tract involvement) (44, 45). Due to that PET scans may detect additional disease sites, the clinical stage is modified in 15% to 20% of patients and therapeutic decision is changed in 8% of patients (46).

Just et al. retrospectively studied 13 HIV-infected patients with BL who underwent one or more PET/CT scans (47). In 5 of 5 patients scanned before treatment, PET/CT demonstrated all involved sites detected at conventional imaging and identified additional sites with high SUVmax in 4 of 5 patients, lymph nodes mainly (4/5), and also spleen (1/5), bone (1/5), and peritoneum (2/5). Lymph node involvement was found in 54% of patients, which is known to be unusual in endemic or sporadic BL. Besides, in 3 patients, BL was predominantly located in the parotid lymph nodes, which is also not a common finding.

As mentioned above, it is a challenge to distinguish HIV-associated lymphoma from benign lymphadenopathy. The likelihood of false positives due to benign lymphadenopathy should be fully taken into consideration when clinicians use FDG PET/CT for initial staging of HIV-associated lymphoma. Patients’ clinical presentations, CD4 counts, imaging features and PET metabolic parameters are helpful to identify the possible cause of lymphadenopathy. However, there are very limited studies on ^18^F-FDG PET/CT for initial staging of HIV-associated lymphoma to date, with small sample sizes. Prospective studies with larger samples are needed in the future.

## 4 PET for response evaluation and prognostic prediction

^18^F-FDG PET/CT has been identified as a reliable technique for both initial staging and early response evaluation in lymphoma, making individualized patient management possible. Previous multicenter studies have found that stage- and risk-adapted treatment is effective and feasible (48, 49). Furthermore, as the 5-year survival rates of both HL and NHL improving, the current interest has shifted towards reducing treatment-related complications (e.g. secondary malignant tumor and cardiovascular events) (50). The ability to promptly and accurately risk-stratify HIV-infected patients with lymphoma will improve the prognosis and decrease the adverse effects of overtreatment.

### 4.1 Baseline PET/CT

The metabolic parameters including SUV, MTV and TLG on baseline PET have been considered as useful predictors of tumor aggressiveness and response to treatment in solid tumors (16, 51). However, the current literatures of HIV-associated lymphoma are somewhat conflicting. Louarn et al. found that all the parameters on baseline PET were associated with progression-free survival (PFS) in univariate analysis, whereas a high total MTV was the only metabolic parameter independently correlated with PFS (Hazard ratio, 3.62) in multivariate analysis. The optimal total MTV cut-off point for prognostic assessment was 527 cm^3^, with a 2-year PFS of 71% (>527cm^3^) compared with 91% (≤527 cm^3^) with p value of 0.004 (52).

On the contrary, Lawal et al. found that the SUVmax, MTV and TLG of lesions were not significantly different between the HIV-positive and HIV-negative groups, whereas presence of HIV infection was related with significantly higher rate of treatment failure following the ABVD (Adriamycin, bleomycin, vinblastine and dacarbazine) regimen compared with the non-infected group (40.4%, 17.7%, respectively, p=0.0034). In univariate analysis, only HIV status of patients was a significant predictor of therapy outcome (p<0.001). SUVmax, MTV and TLG of lesions as well as the Ann Arbor stage were not significant in predicting therapy outcome. Furthermore, a multiple logistic regression showed that HIV status alone was significant in predicting therapy outcome [overall rate (OR)=2.930, 95% confidence interval (CI), 1.197-7.172, p=0.023] among the metabolic parameters and Ann Arbor stage (53).

These findings were supported by another study by the same author. Lawal et al. analyzed 160 patients with HL, including 57 HIV-positive patients. The median values of SUVmax, SUVmean, MTV and TLG were slightly higher among the HIV-positive group compared with the HIV-negative group. However, no significant difference was found between the two groups. In addition, among the seven parameters of International Prognostic Score (IPS) indicating poor prognostic factors, only male sex (HIV-negative group higher, p=0.005) and serum albumin less than 4 g/dl (p=0.009) were found with significant differences between the two groups (54).

In paediatric and adolescent patients with HIV-associated lymphoma, similar results were found in a retrospective study. Reed et al. found that HIV status and treatment response on PET were significantly related to PFS (p=0.036, p<0.001, respectively) in univariate analysis. In contrast, none of the metabolic parameters was significantly predictive of either PFS or overall survival (OS). Unlike in the adult research, the baseline total MTV was considered as a significant predictor of treatment response (p=0.017) (55).

### 4.2 Interim PET/CT

Interim ^18^F-FDG PET/CT in both HL and NHL has demonstrated prognostic significance in the general population: residual tumor avidity on PET after 2 to 4 cycles corresponds with lower PFS, while interim PET negative is correlated with higher PFS (56–58). Early identification of patients at high risk of standard treatment failure will affect treatment decisions (more intensive therapy) and ultimately improve patient treatment outcomes and survival.

However, sparse studies exist about the prognostic value of interim ^18^F-FDG PET/CT in patients with HIV-associated lymphoma. Okosun et al. showed that interim PET negativity was significantly associated with a higher PFS. A total of 23 patients with advanced HIV-associated HL were included in this analysis. Deauville criteria was used to evaluate the metabolic activity of lesions, and a Deauville score of 1-3 was defined as PET-negative. After a median follow-up of 27 months (range, 12-50 months), the 2-year PFS rates for interim PET-positive and PET-negative patients were 50% and 100% respectively (log-rank p=0.0012) (59).

A similar result of relationship between interim PET findings and OS was reported by Minamimoto, R. et al. (60). A total of 24 patients with HIV-related malignant lymphoma (13 DLBCL, 11 BL) were included in this study, who underwent interim PET/CT. In 10 of 24 cases, interim PET findings were evaluated as “positive”, while the rest cases were evaluated as “negative”. Interim PET negativity was associated with significantly longer OS (932 ± 549 days) compared to positive cases (454 ± 442 days, p=0.043). Over all two year survival rate of negative findings on interim PET was 80% (95%CI, 69%-91%), which was higher than 29% (95%CI, 16%-41%) in positive cases. Moreover, Cox regression analysis showed strong prognostic influences of interim PET findings (Hazard ratio 4.57, 95%CI 0.88-23.73, p=0.07) and Eastern Cooperative Oncology Group performance status (Hazard ratio 10.52, 95%CI 1.26-87.82, p=0.03) on OS.

A phase 2 trial implied that response-adapted therapy based on interim PET was feasible in patients with HIV-associated HL (61). All patients underwent interim PET scan after 2 initial cycles of ABVD, and 10 of 12 patients achieved PET-negative status, while 2 of 12 remained PET-positive (Deauville scores 4 or 5) according to Deauville criteria. All the PET-negative and one PET-positive patients continued ABVD regimen. The other PET-positive patient received 6 cycles of BEACOPP (bleomycin, etoposide, doxorubicin, cyclophosphamide, vincristine, procarbazine and prednisone) regimen. Finally, 75% of HIV-HL patients achieved complete response (CR) and 25% partial response (PR). With a median follow-up of 39 months (range, 5-53 months), a 2-year PFS was 83% (95% CI, 46.1%-95.3%), which was similar to that in non-HIV-HL patients in the same phase 2 trial (2-year PFS, 79%) (62). The prognostic predictions of both baseline and interim FDG PET for HIV-associated lymphoma are summarized in **Table 2**.

**Table 2:** The prognostic predictions of both baseline and interim FDG PET for HIV-associated lymphoma.

PET imaging	References	Study type	Number of patients	Subtypes	Parameters	Cut-off value	P value	AUC	Complete remission (%)	Survival (%)
**Baseline PET**	52	Retrospective	109, HIV, +	HL	tMTV	≤527	**0.004**	0.60	87	2-year PFS 915-year OS 89.1
						>527				2-year PFS 715-year OS 69.4
					TLG	≤230	NS	0.59		5-year OS 96.0
						>230				5-year OS 82.9
					SUVmax	≤8.7	NS	0.55		5-year OS 94.4
						>8.7				5-year OS 81.1
					SUVpeak	≤7.1	NS	0.55		5-year OS 94.6
						>7.1				5-year OS 80.8
					SUVmean	≤5.1	NS	0.53		5-year OS 94.3
						>5.1				5-year OS 81.1
	53	Prospective	136 (57, HIV, +)	HL	SUVmax	NA	0.087	NA	NA	NA
					MTV	NA	0.589	NA		NA
					TLG	NA	0.460	NA		NA
	54	Retrospective	160 (57, HIV, +)	HL	SUVmax	NA	0.401	NA	NA	NA
					SUVmean	NA	0.056	NA		NA
					MTV	NA	0.766	NA		NA
					TLG	NA	0.965	NA		NA
	55	Retrospective	69 (13, HIV, +)	HL	SUVmax	NA	0.379	NA	58	NA
					MTV	NA	0.065	NA		NA
					TLG	NA	0.099	NA		NA
					total MTV	NA	**<0.001**	NA		NA
**Interim PET**	59	Retrospective	23, HIV, +	HL	PET negative	NA	**0.0012**	NA	96	2-year PFS 100
					PET positive	NA				2-year PFS 50
	60	Retrospective	24, HIV, +	13 DLBCL 11BL	PET negative	NA	**0.043**	NA	NA	2-year OS 80
					PET positive	NA				2-year OS 29
	61	phase 2 tiral	12, HIV, +	HL	PET negative	NA	NA	NA	75	2-year PFS 83
					PET positive	NA	NA	NA		

Statistically significant values are highlighted in bold.

1. AUC, area under the curve; NA, not available; 2. HL, Hodgkin’s lymphoma; DLBCL, diffuse large B-cell lymphoma; BL, Burkitt’s lymphoma; 3. SUV, standard uptake value; SUVmax, the maximum of SUV; SUVmean, the mean of SUV; MTV, metabolic tumor volume; TLG, total lesion glycolysis; 4. PFS, progression-free survival; OS, overall survival.

## 5 PET-guided radiotherapy decision

The success of an individualized treatment decision depends largely on accurate staging and response assessment. Engert A et al. have suggested that PET done after chemotherapy was helpful to guide the need for additional radiotherapy in patients with HL lymphoma (63). In advanced-stage HL, patients with a persistent mass after chemotherapy measuring 2.5 cm or larger and positive on PET scan should receive additional radiotherapy. For patients with nonbulky stage I/II DLBCL and negative on interim PET scan, chemotherapy alone was comparable to chemotherapy followed by radiotherapy; additional radiotherapy was applied to patients with positive on interim PET scan (64, 65).

## 6 PET at the end of therapy

FDG-PET plays an important role in the response assessment of both HL and NHL at the end of therapy, especially in the identification of a CT-detected residual mass (16). An FDG-PET/CT scan after completion of the intended treatment can differentiate viable tumor cells from fibrosis or necrosis. Engert A et al. reported that post-treatment PET scans were able to sharply reduce the number of patients with additional radiotherapy for residual mass to 11% from 71% in previous trials (66). The Deauville 5-point scale is used to assess differing degrees of response at the end of treatment (67). A Deauville score of 1 to 3 identifies complete metabolic response, while residual disease is defined by a score of 4 to 5.

## 7 Surveillance PET scans

Relapses occur in approximately 10-20% of patients with early stage HL, as well as 30-40% in those at advanced stages following first-line therapies (68). There is sparse available published literature of relapsed HIV-associated lymphoma. In general population, the role of FDG PET in post-treatment surveillance remains controversial.

In a prospective study, 5 patients were detected relapsed or refractory disease by FDG PET, while 6 patients were found to have false-positive findings (69). These findings are corroborated by a number of other studies. A multicenter retrospective study of 161 patients with HL showed that the overall positive predictive value (PPV) and negative predictive value (NPV) of PET were 28% and 100% respectively (70). In a retrospective analysis of 75 patients with DLBCL, the PPV of PET was 85%, but usefulness was only for high-risk patients with symptoms indicative of a relapse and those older than 60 years (71).

Thus, there is currently insufficient evidence to recommend PET as a routine surveillance tool for patients with lymphoma. FDG PET should be used only for patients at a high-risk of recurrence to reduce radiation burden and costs.

## 8 A simple comparison of the role of FDG PET in COVID-19 and HIV pandemic

A systemic review was conducted to evaluated the role of ^18^F-FDG PET/CT in patients with Coronavirus Disease (COVID-19) (72). In 10 of 11 studies, FDG PET/CT was used to assess the oncological indications and incidental findings suspicious of COVID-19 infection, particularly in suspected COVID-19 interstitial pneumonia. Only in one study, FDG PET/CT was performed to evaluate the inflammatory status at the presumed peak of the inflammatory phase. Evidence-based data has demonstrated an increased incidence of FDG PET/CT abnormalities evocative of a pulmonary infection in asymptomatic patients during the COVID-19 outbreak. However, it should be noted that FDG PET cannot substitute or integrate high-resolution CT to diagnose suspicious COVID-19 or for disease monitoring, which is different from the broader role of FDG PET in patients with HIV-associated lymphoma.

## 9 Conclusions and future perspectives

Taken together, PET metrics including SUV, SUL, MTV, and TLG were the most frequently observed significant parameters in the studies for differential diagnosis, response evaluation and prognostic prediction of HIV-associated lymphoma. Furthermore, baseline, interim and post-treatment PET imaging findings have been found reliable to guide clinical decision, which ultimately improves treatment outcomes and survival.

Nevertheless, the majority of current studies were single-center, retrospective studies with limited sample sizes. Additionally, the rapid development of radiomics and PET/MR will provide further lesion features and improve the PPV of PET imaging. To better understand the ability of increasingly novel radiotracers of PET imaging in HIV-associated lymphoma, including ^68^Ga-FAPI and biomarkers of immunoPET, prospective studies with large populations and multicenter are necessary.

## Author contributions

QL and TY wrote the essay. TY and YL revised the introduction and the first half of the article. XC helped to revise the framework of the article. All authors contributed to the article and approved the submitted version.
